# Population of Endogenous Pararetrovirus Genomes in Carrizo Citrange

**DOI:** 10.1128/genomeA.01063-13

**Published:** 2014-01-16

**Authors:** Avijit Roy, Jonathan Shao, William L. Schneider, John S. Hartung, Ronald H. Brlansky

**Affiliations:** a University of Florida, IFAS, Plant Pathology Department, Citrus Research and Education Center, Lake Alfred, Florida, USA; b USDA-ARS, FDWSRU, Ft. Detrick, Maryland, USA; c USDA-ARS, Beltsville Agricultural Research Center, Molecular Plant Pathology Laboratory, Beltsville, Maryland, USA

## Abstract

The complete genome sequences of three related endogenous pararetroviruses (EPRVs) were obtained by 454 sequencing of nucleic acid extracts from Carrizo citrange, used as a citrus rootstock. Numerous homologous sequences have been found in the sweet orange genome. The new EPRVs are most closely related to petunia vein-clearing virus.

## GENOME ANNOUNCEMENT

Genomes related to those of members of the *Caulimoviridae* family may be found to be integrated in plant genomes. Many are silent, but some may replicate and cause viral infection under certain conditions (1). These endogenous pararetrovirus (EPRVs) have been reported to cause diseases, including banana streak virus (2), tobacco vein-clearing virus (3), dahlia mosaic virus (D10) (4), cassava vein mosaic virus (5), and petunia vein-clearing virus (PVCV) (6). EPRVs can be induced to replicate by hybridization, tissue culture, heat, drought stress, and wounding (7). Their double-stranded circular genomes contain single-strand nicks, range in size from 7,206 bp (for PVCV) to 8,158 bp (for cassava vein mosaic virus), and have from 1 to 5 open reading frames (ORFs).

Carrizo, a hybrid of a Washington navel sweet orange and *Poncirus trifoliata*, is a widely used rootstock for citrus production. Mature roots of Carrizo rootstocks from healthy sweet orange trees were collected in a full-grown commercial orange grove in Polk County, Florida. The bark was removed and used to obtain a preparation enriched with phloem cells. The tissue was then flash-frozen and ground to a powder in liquid nitrogen. RNA from the frozen extract was then purified using a TRIzol-based protocol (Life Technologies). The RNA-enriched nucleic acid preparation was then used as template for 454 sequencing. Sixty-five thousand reads were obtained, trimmed, and assembled into 575 contigs that were used to search the NCBI database. BLASTn returned very significant matches to PVCV, with E values ranging from 7E−109 to 0. These PVCV-like contigs were aligned and primers were designed to amplify the remaining sequences by conventional and inverse PCR using DNA-enriched nucleic acid preparations as a template. The complete viral genomes were assembled using Sanger sequencing of cloned PCR amplicons.

Three similar EPRVs were obtained, with genomes of 6,663, 6,957, and 6,996 nucleotides (nt). Each of these genomes has 2 ORFs. ORF1 was preceded by the sequence encoding the tRNA methionine binding site. The 5′ end of ORF1 has the greatest amount of variation in sequence. Deletions and insertions occurred, but the reading frame was maintained. The length of ORF1 varies from 5,106 to 5,400 nt and encodes a protein with the signature domains of the PVCV polyprotein, including the movement protein, zinc finger, reverse transcriptase-with long terminal repeat, RNA-dependent RNA polymerase, and RNAse H regions. An integrase domain was not observed (6). ORF2 is 417 nt in all three genomes, but no matches for the 139 amino acid hypothetical proteins (p15) were found at NCBI. Bioinformatics revealed many related sequences in the sweet orange genome, with sequence identities of 85 to 89% to the three EPRVs. Sequences with homology to PVCV ORF1 have been reported previously in the citrus tristeza virus (CTV) resistance locus of *P. trifoliata* (8), one of the parents of Carrizo. These virus sequences are being analyzed in citrus trees affected with the citrus decline disease citrus blight.

### Nucleotide sequence accession numbers.

The Carrizo EPRV sequences have been deposited in Genbank as accession no. KF800043, KF800044, and KF800045.
